# Small Force, Big Impact: Next Generation Organ-on-a-Chip Systems Incorporating Biomechanical Cues

**DOI:** 10.3389/fphys.2018.01417

**Published:** 2018-10-09

**Authors:** Ece Ergir, Barbara Bachmann, Heinz Redl, Giancarlo Forte, Peter Ertl

**Affiliations:** ^1^Center for Translational Medicine, International Clinical Research Center, St. Anne’s University Hospital, Brno, Czechia; ^2^Faculty of Technical Chemistry, Institute of Applied Synthetic Chemistry and Institute of Chemical Technologies and Analytics, Vienna University of Technology, Vienna, Austria; ^3^AUVA Research Centre, Ludwig Boltzmann Institute for Experimental and Clinical Traumatology, Vienna, Austria; ^4^Austrian Cluster for Tissue Regeneration, Vienna, Austria; ^5^Kompetenzzentrum für MechanoBiologie (INTERREG V-A Austria – Czech Republic Programme, ATCZ133), Vienna, Austria; ^6^Competence Center for Mechanobiology (INTERREG V-A Austria – Czech Republic Programme, ATCZ133), Brno, Czechia; ^7^Department of Biomaterials Science, Institute of Dentistry, University of Turku, Turku, Finland

**Keywords:** microfluidics, mechanobiology, organ-on-a-chip, lab-on-a-chip, *in vitro* organ models, mechanical cell actuation

## Abstract

Mechanobiology-on-a-chip is a growing field focusing on how mechanical inputs modulate physico-chemical output in microphysiological systems. It is well known that biomechanical cues trigger a variety of molecular events and adjustment of mechanical forces is therefore essential for mimicking *in vivo* physiologies in organ-on-a-chip technology. Biomechanical inputs in organ-on-a-chip systems can range from variations in extracellular matrix type and stiffness and applied shear stresses to active stretch/strain or compression forces using integrated flexible membranes. The main advantages of these organ-on-a-chip systems are therefore (a) the control over spatiotemporal organization of *in vivo*-like tissue architectures, (b) the ability to precisely control the amount, duration and intensity of the biomechanical stimuli, and (c) the capability of monitoring in real time the effects of applied mechanical forces on cell, tissue and organ functions. Consequently, over the last decade a variety of microfluidic devices have been introduced to recreate physiological microenvironments that also account for the influence of physical forces on biological functions. In this review we present recent advances in mechanobiological lab-on-a-chip systems and report on lessons learned from these current mechanobiological models. Additionally, future developments needed to engineer next-generation physiological and pathological organ-on-a-chip models are discussed.

## Introduction

**FIGURE 1:**
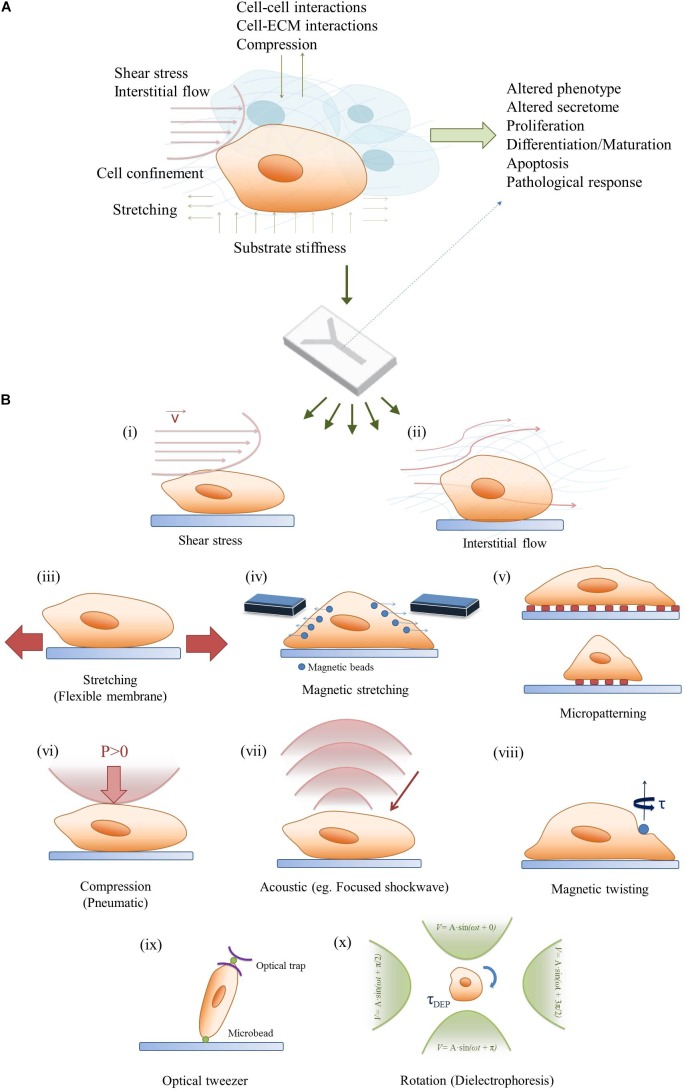
Bridging the *in vivo/in vitro* gap in mechanobiology. **(A)** A combination of mechanobiological cues in the microenvironment can regulate cell signaling and phenotype as well as physiological and pathological tissue response. **(B)** A simplified demonstration of a mechanobiology-on-a-chip, and potential on-chip stimulation strategies for microfluidic 2D/3D cell cultures: (i) Shear stress, (ii) Interstitial flow, (iii) Stretching, (iv) Magnetic stimulation, (v) Micropatterning, (vi) Compression, (vii) Acoustic stimulation, (viii) Magnetic twisting, (ix) Optical tweezers, and (x) Rotation (dielectrophoresis).

Conventional mammalian cell culture systems are predominantly based on two-dimensional (2D) monolayer cultures to study cellular mechanisms in pharmaceutical research, toxicology and biomedicine. Even though 2D cell culture models are routinely used, they fail to recapitulate the spatiotemporal dynamics that cells encounter *in vivo*. The discrepancy between *in vitro* monolayers and native tissue structures leads in many cases to altered phenotype, cell morphology and behavior, thus rendering results from 2D cell-based assays questionable (Baker and Chen, 2012). To overcome the drawbacks associated with 2D monocultures, recent cell cultivation setups aim to re-engineer the physiological cellular microenvironment of native tissues. This can be accomplished by moving toward three-dimensional (3D) culture systems through cultivating cells in hydrogels, scaffolds or aggregates (Chen et al., 2002; Drury and Mooney, 2003; Griffith and Swartz, 2006; Fennema et al., 2013). The implementation of 3D cell culture systems by itself allows for indirect mechanical stimulation by controlling the rigidity and stiffness of the extracellular matrix, which has shown to modulate cellular responses (Engler et al., 2006; Kumar, 2014). Additionally, 3D-tissue models can be further subjected to dynamic mechanical stimuli including fluid flow, stretch/strain and compression. The application of indirect and/ or direct mechanical stimuli in tissue cultures has become a rapidly growing research field that is commonly known as mechanobiology (Eyckmans et al., 2011). It is important to highlight that almost every tissue of a human body is subjected to either constant or temporary mechanical stimuli. This means that the application of mechanobiological forces represents a vital step toward the establishment of physiological microenvironments *in vitro* (Wang and Thampatty, 2006; Jansen et al., 2015). To date a variety of mechanobiological forces, shown in **Figure 1A**, have been employed in 2D and 3D *in vitro* cultures including (a) matrix stiffness that mimic the respective Young’s moduli of native tissue, (b) fluid flow in vascular systems and interstitial tissue, (c) stretch/strain mechanisms in the lung, heart and gastrointestinal tract as well as (d) compression in the musculoskeletal system (Lovett et al., 2009; Riehl et al., 2012; Shachar et al., 2012; Ahearne, 2014). These mechanobiological systems demonstrated improved cell-to-cell and cell-to-matrix interactions resulting in significant progress in recapitulating physiological microenvironments *in vitro*. Although these tissue models led to substantial advances in understanding mechanobiology on a macroscale, common tissue engineering-based approaches require sizeable amounts of cells as well as reagents but lack precise control over location and amount of the stimulus. A commonly accepted solution in fostering our understanding of biomechanical effects is taking the tissue to the microscale. This can be accomplished by mimicking cellular microenvironments in microfluidic devices, which not only offer a decrease in cell numbers and consumables but also allow precise control over spatio-temporal stiffness and growth factor gradients as well as mechanical stimulus type, amount and location (Huh et al., 2011; Bhatia and Ingber, 2014; Rothbauer et al., 2015; van Duinen et al., 2015). In other words, microdevices can be used to investigate contractility, cell confinement and micropatterning, all of which are crucial in gaining deeper insights into mechanobiological phenomena. Moreover, microfluidic chips are compatible with high resolution microscopy for cell observation and also allow the integration of actuators and sensors, which provides the opportunity to trigger and monitor cellular behavior *in situ*. Overall this review focuses on emerging physiologically relevant micro-tissue models in mechanobiology-on-a-chip setups in both culture environments since 2012, shortly summarized in **Table 1**.

**Table 1:** Summary on recent reports on mechanobiological approaches for cell manipulation in microfluidic devices.

Biomechanical stimulus	Organ culture	Cell type	Environment	Reference
Interstitial flow	Vasculature	Primary	3D	Hsu et al., 2013; Jeon et al., 2014; Kim S. et al., 2016
Interstitial flow	Brain	Primary	3D	Park et al., 2015; Wang et al., 2018
Interstitial flow	Liver	Primary	3D	Lee et al., 2013
Substrate stiffness	-	Cell line	2D	García et al., 2015
Electromechanical	-	Primary	2D	Pavesi et al., 2015
Shear stress	Vasculature	Primary	2D	Charwat et al., 2018
Shear stress	-	Cell line	2D	Soffe et al., 2017
Shear stress	Blood brain barrier	Cell line	2D	Griep et al., 2013
Shear stress	Aortic valve	Primary	2D	Wang et al., 2017
Shear stress	Blood brain barrier	Primary	2D/3D	Brown et al., 2015
Shear stress	Blood brain barrier	Cell line	2D/3D	Sellgren et al., 2015
Shear stress	Extravasation	Primary	3D	Jeon et al., 2015
Shear stress	Vasculature	Primary	3D	Kim et al., 2013
Shear stress	Bone	Cell line	2D	Middleton et al., 2017a,b
Shear stress	Bone	Primary	3D	Altmann et al., 2014
Shear stress	Vasculature	Primary	2D	Zheng et al., 2017
Shear stress	Vasculature	Primary	2D	Venugopal Menon et al., 2018
Shear stress	Vasculature	Primary	2D	Patibandla et al., 2014
Stretching	Lung	Primary and cell line	2D	Huh et al., 2010, 2012; Benam et al., 2016; Hassell et al., 2017; Jain et al., 2018
Stretching	Gut	Primary and cell line	2D	Kim H.J. et al., 2016; Villenave et al., 2017
Stretching	Heart	Primary	2D	Ugolini et al., 2016, 2017
Stretching	Muscle	Primary and cell line	2D	Michielin et al., 2015
Stretching	Vasculature	Primary	2D	Zhou and Niklason, 2012
Stretching	-	Primary	3D	Liu et al., 2016
Stretching	Heart	Primary	3D	Marsano et al., 2016; Occhetta et al., 2018
Stretching	-	Cell line	3D	Li et al., 2016
Stretching	-	-	2D/3D	Gizzi et al., 2017
Stretching	Artery	Primary	2D	van Engeland et al., 2018
Compression	Bone	Primary	2D	Park et al., 2012
Compression	Vasculature	Primary	2D	Sticker et al., 2017

*The dashes (-) identify studies with devices without a defined target organ, tissue or cell culture*.

## Microfluidic Mechanobiology in Monolayers and Barrier Models

### Shear Stress

Microfluidic devices with their laminar flow regimes have widely been used to expose cells to fluid flow induced shear stress. This has led to substantial improvements in understanding the mechanobiological effect of shear stress variations on endothelial cells in vascular models but also in osteocyte, cardiomyocyte and epithelial cell biology. In particular, fluid flow plays a major role in vascular biology as the endothelial cell lining layer inside blood vessels is constantly exposed to pulsatile blood flow (Baratchi et al., 2017). Subjecting endothelial cells to physiological unidirectional or disturbed shear stress patterns has been shown to significantly alter cell morphology and phenotype. For example, it has been shown that shear stress influences nanoparticle uptake of endothelial cells where higher flow rates led to reduced uptake. Using an *in vitro* as well as *in silico* approach, Charwat et al. (2018) found that clathrin-mediated uptake of nanoparticles is drastically reduced when exceeding shear forces of 1.8 dyn/cm^2^, implying an important role of shear stress when investigating *in vitro* nanoparticle uptake. Another study, published by Griep et al. (2013), successfully recreated the smallest unit of the blood brain barrier using immortalized brain endothelial cells to study barrier integrity in the presence of physiological shear force. While unidirectional shear stress plays an important role in assessing healthy vessel physiology, microfluidic devices can also be utilized to create bi- and multidirectional flow patterns for mimicking endothelial pathology. Such disturbed flow patterns allow for flow type-dependent gene expression profiling of endothelial cells (Zheng et al., 2017) as well as observation of leucocyte – endothelial cell interactions (Venugopal Menon et al., 2018) and the role of glucose uptake in endothelial dysfunction (Patibandla et al., 2014) for modeling inflammation and hyperglycemia in atherosclerosis. The effect of shear stress on endothelial cells and microfluidic technologies have recently been extensively reviewed elsewhere (Smith and Gerecht, 2014; Haase and Kamm, 2017; Kim et al., 2017). Furthermore, microfluidic devices have also been used outside of endothelial research to record phenotypic transformations of aortic valve interstitial cells during applied shear stress and to monitor morphological changes of osteocytes during application of flow. Additionally, Middleton et al. (2017a) showed that in the presence of shear stress, cell to cell interactions of osteocytes co-cultured with osteoclast are enhanced, leading to the improved mechanical response of bone cells. A more detailed review on monitoring cell-cell interaction using microfluidic devices was recently published by Rothbauer et al. (2017). Altogether, microfluidic devices are an important tool to study shear stress, but while the effects on endothelial cells have been studied extensively, other cell types and different co-culture models would certainly benefit from further research.

### Stretching

In contrast to shear-dependent mechanobiological effects, microfluidic devices have recently come into focus for their ability to engineer miniaturized functional barrier units. Different from the aforementioned models where cells are grown in monolayer in microchannels, these systems aim to recreate the smallest possible unit of an organ by mimicking the barrier between two monolayers. An overview of such a microfluidic device including the respective cell actuation can be found in **Figure 1B**. The cells are cultivated back-to-back on cyclically stretched flexible membranes emulating organotypic movements. With the most famous example still being the Lung-on-a-Chip published by Huh et al. (2010), a number of similar systems have since been used to investigate a variety of different barrier models. For example, using the same microdevice containing a flexible membrane that is stretched by applying vacuum to two air channels on either side of the cultivation chamber, different pathological scenarios have been recapitulated, including pulmonary edema (Huh et al., 2012), small-airway inflammation (Benam et al., 2016), orthotopic lung cancer extravasation, growth and therapy (Hassell et al., 2017) and intravascular thrombosis assessment (Jain et al., 2018). A similar device has also been used to mimic bacterial overgrowth, inflammatory bowel disease (Kim H.J. et al., 2016) and virus infection (Villenave et al., 2017) in a gut-on-a-chip system and to study the effect of cyclic strain on proliferation and adaptive responses of cardiac fibroblasts (Ugolini et al., 2016, 2017) and to study endothelial cell and smooth muscle cell signaling under hemodynamic loading (van Engeland et al., 2018). A different design approach to exert stretch/strain forces in microfluidic cell cultures employ pressurized air to deflect a membrane on which cells are cultured. Cyclic mechanical actuation of a myoblast cell line and primary myoblasts using such a device is demonstrated in a muscular dystrophy model (Michielin et al., 2015) and to recreate the cyclic strain of blood vessels (Zhou and Niklason, 2012). While most examples in the literature focus on uniaxial stretching, a computationally informed, vacuum-actuated multi-axial microfluidic chip device has recently been developed that allows programmable actuation along different directions (Gizzi et al., 2017), further advancing the opportunities for mechanobiological on-chip investigations from the broadly used uniaxial strain barrier model devices.

### Compression and Other Novel Methods

A relatively new approach for mechanobiological stimulation in microfluidic devices is cellular compression. Even though this has been shown to stimulate the osteogenic differentiation of stem cells (Park et al., 2012) and used to investigate wound healing (Sticker et al., 2017), further studies need to be conducted to improve our understanding of compression-based biomechanical stimuli. Other methods to incorporate mechanobiological signals in microchips exploit the fact that microfluidics is ideally suited to create and monitor spatiotemporal gradients. For example, Soffe et al. (2017) showed that human embryonic kidney cells respond to shear stress gradients using trapezoid microchannel geometries, while García et al. (2015) investigated the effects of substrate stiffness gradients on cell behavior (Soffe et al., 2017). A sophisticated approach is reported by Pavesi et al. (2015): cells were subjected to multiple mechanical stimuli by adding the possibility of simultaneous electrical stimulation. The dual-stimulation strategy led to morphological and phenotypical cellular changes as well as altered cytoskeletal fiber orientation in mesenchymal stem cells. Nonetheless, while all the mentioned studies report an increase in physiological behavior of the cultured cells upon the exposure to mechanobiological cues, recreating of physiological microenvironment in a two-dimensional cultivation setup remains challenging.

## Microfluidic Mechanobiology in the Third Dimension

Even though the above described microfluidic 2D models seem suitable for recreating lining layers and barrier models, they still do not resemble physiological tissue architecture since most cells reside in a three-dimensional tissue matrix in their native environment. Hence, the third dimension remains an important issue that needs to be considered when aiming to re-engineer organ models *in vitro.* It is important to note that the physiological tissue microenvironment is composed of a variety of complex physical properties ranging from cell-cell interactions to the extracellular matrix composition and biomechanical stimuli such as dynamic stretching and compression. This biological complexity, to date, poses a significant challenge, since only a limited number of studies incorporating 3D mechanobiology on microfluidic chips have been reported.

### Interstitial Flow

Additional to utilizing microfluidic devices in endothelial biology for investigating the effect of shear stress on monolayers, microchips are routinely used to determine the effects of interstitial flow on 3D vasculo- and angiogenesis. One approach involves the combination of vasculature with fluid flow where endothelial cells are co-cultured with supporting mural cells in hydrogels to generate blood vessels via vasculogenesis or angiogenesis. Hsu et al. (2013) developed a microfluidic device that is based on a resistive circuit concept to create an array of vascularized microtissue chambers. Interstitial fluid flow in the physiological range of 0.5 to 10 μm/s showed enhanced vessel-like structure formation corroborating similar studies from Jeon et al. (2014) for blood angiogenesis and from Kim S. et al. (2016) for lymphangiogenesis (Hsu et al., 2013). A more detailed review of recent advances in on-chip vascularization was recently published by Haase and Kamm (2017). However, microfluidics not only provides the perfect tool for exploring the importance of interstitial flow in endothelial cell biology but also for bone and brain organ-on-a-chip devices. In a recent study by Altmann et al. (2014), the morphogenesis of 3D cultured human primary alveolar bone osteoblasts under static and microfluidic growth conditions was compared. The cells were allowed to form aggregates in 300 μm cavities with fibronectin coating in poly (methyl methacrylate)-based chips and exposed to perfusion of 15, 30, and 60 μl/min flow rates. It was found that fluid flow lead to more distinct morphogenesis and more bone-specific gene expression and extracellular matrix formation after 7 days of culture. Additionally, Park et al. (2015) reported the design of a microfluidic chip-based 3D neurospheroid culture consisting in concave microwell arrays in which interstitial flow was generated by an osmotic micropump system. The results of the study showed that when neurospheroids are cultured under flow conditions, larger and more complex neural networks were formed compared to static culture. In a more recent 3D brain model, human induced pluripotent stem cell derived organoids were integrated into a microdevice using three-dimensional Matrigel. The on-chip cultured organoids showed improved neural differentiation and cortical organization under perfusion culture as well as enhanced expression of cortical layer markers, thus demonstrating the importance of 3D culture and mechanical fluid flow in enhancing brain organogenesis (Wang et al., 2018). Furthermore, Brown et al. (2015) created a complex physiologically relevant blood-brain-barrier model based on a back-to-back culture of endothelial cells that mimic blood vasculature with a co-culture of astrocytes, pericytes and hydrogel-embedded neurons that showed improved tight-junction formation under fluid flow conditions. These examples show that interstitial flow plays a crucial role for recreating physiological microenvironments and call for further research including a variety of other organ models.

### Stretching and Compression in 3D

Other than the sophisticated stretch/strain devices for monolayer barrier models, compression and stretching of 3D microfluidic organ models is still in its infancy. One of the first applications involve a 3D cell construct that is exposed to cyclic mechanical strain to develop a beating heart-on-a-chip (Marsano et al., 2016). In this study, human induced pluripotent stem cell-derived cardiomyocytes were embedded in a fibrin gel prior to injection into the microdevices, and subsequently exposed to mechanical stimulation using a deformable PDMS (Polydimethylsiloxane) membrane (10% uniaxial strain, 1 Hz frequency). Interestingly, the mechanically stimulated constructs showed similar gene expression levels of cardiac markers when compared to non-actuated controls. However, the mechanical stimulus resulted in decreased expression of MYH6 (a marker for less developed phenotype), thus indicating superior cardiac maturity compared to static conditions. Furthermore, elongated cardiac-like morphology was observed in the mechanically stimulated constructs. Recently, the same group employed a similar concept to propose a model of cardiac fibrosis by applying cyclic mechanical stretch to cardiac fibroblasts embedded in a 3D fibrin hydrogel. By exploiting this strategy, the authors claim to be able to mimic some of the key steps of cardiac fibrosis onset in a timely fashion: early fibroblast proliferation, their phenotype switch into myofibroblasts, extracellular matrix deposition and its final stiffening (Occhetta et al., 2018). An alternative microfluidic device containing deformable membranes was developed to investigate differentiation associated matrix production using a real-time stiffness sensor. The authors showed that mesenchymal stromal cells embedded in hydrogels and subjected to dynamic mechanical stimulation undergo myofibroblast differentiation and synthesize collagen, leading to gel stiffening (Liu et al., 2016). In another study, 3D responses of cells were quantified in the presence of extreme strain within 3D hydrogel matrices. Here, micro-magnetically actuated synthetic tissue cultures were developed and consisted of a polyethylene glycol dimethacrylate hydrogel layer containing iron microspheres, and a stiffness tunable gelatin methacryloyl hydrogel containing a population of fibroblasts. Using this magnetic field focusing device, strain-dependent proliferation, spreading, polarization, differentiation, and matrix adhesion was studied (Li et al., 2016). Although the system can be used to readily adjust mechanical strain within 3D hydrogel cell cultures, some limitations remain concerning extracellular matrix porosity and non-fibrous matrices not being representative of the cell environment in a real tissue. **Table 1** lists currently available technologies according to applied stimuli, organ culture and cell type using microfluidic 2D as well as 3D cell culture systems.

## Concluding Remarks and Further Perspectives

The combination of microfabrication-based technologies with complex biology has enabled the development of advanced *in vitro* models capable of culturing and analyzing cell and tissue constructs under physiologically relevant conditions (Ertl, 2015; Rothbauer et al., 2015, 2017). While microfluidic models for 2D mechanical stimulation involving stretch and strain has been widely investigated, the application of physiologically relevant axial strain in 3D cell culture systems is still in its infancy. To date only few microdevices have been developed that are capable of recreating mechanobiological relevant three-dimensional cellular microenvironments. Current mechanobiology-on-a-chip advances are hindered by both technological shortcomings and the limited reliability of current *in vitro* 3D cell culture systems. One possible solution to improve fabrication speed, precision, material selection, and (bio)compatibility could be stereolithography, which already enables (a) additive manufacturing of microchannels down to 300 μm and (b) the integration of pneumatic valves for automated cell handling and manipulation of complex biological structures on chip. Next generation microfluidic devices will need to contain computer-controlled valves and micropumps for fluid-mechanical stimulation of cells (Rogers et al., 2015; Chen et al., 2016) and integrated actuators to reliably regulate and modify mechanical forces on tissue constructs. Furthermore, future microfluidic devices will need to address current limitations in microdevice operation to minimize the need for bulky off-chip equipment such as pumps, heaters, microscope, gas-supply, and connectors. While the incorporation of micropumps and -valves or alternative approaches for on-chip fluid handling have already been demonstrated in state-of-the-art devices (Sung et al., 2010; Kim et al., 2012; Hasenberg et al., 2015), integrated sensing solutions that replace off-chip detection methods are still scarce. The integration of micro- and nanosensors will ultimately enable the investigation of dynamic cellular responses to any imaginable physical, chemical and biological stimuli, thus providing detailed information on tissue behavior down to the molecular level. In conclusion, given the complexity of *in vivo* biological architectures of tissues and organs, next generation mechanobiology-on-a-chip systems will need to significantly increase the similarity of *in vitro* 3D biologically inspired constructs using highly integrated, fully automated and miniaturized cell analysis systems.

## Author Contributions

EE and BB conceived the general structure of the review, revised the existing literature, and drafted the manuscript. HR, GF, and PE revised the text and contributed to the final manuscript.

## Conflict of Interest Statement

The authors declare that the research was conducted in the absence of any commercial or financial relationships that could be construed as a potential conflict of interest.
